# Psychometric properties of the two-item Pittsburgh Sleep Quality Index (PSQI-2) in a cohort of community-dwelling older men

**DOI:** 10.21203/rs.3.rs-7526287/v1

**Published:** 2025-10-20

**Authors:** Luiz Antônio Alves de Menezes-Júnior

**Affiliations:** Federal University of Ouro Preto

**Keywords:** Sleep Quality, Validation Studies, Geriatrics, Reproducibility of Results, Epidemiologic Methods

## Abstract

**Background:**

The full Pittsburgh Sleep Quality Index (PSQI) is a widely used measure of sleep quality, but can be impractical in large studies due to its length. The abbreviated two-item version (PSQI-2) is a promising alternative, yet its longitudinal psychometric properties remain underexplored in community-based cohorts of older adults.

**Objective:**

To comprehensively evaluate the cross-sectional and longitudinal validity of the PSQI-2 against the full PSQI in a cohort of older men.

**Methods:**

This longitudinal analysis utilized data from 2,911 participants in the Osteoporotic Fractures in Men (MrOS) Sleep Study with complete sleep data at two visits. Cross-sectional validity was assessed using mixed-effects regression and Bland-Altman analysis. Diagnostic accuracy for poor sleep quality (PSQI > 5, >7, and > 10) was evaluated with Receiver Operating Characteristic (ROC) curves. Longitudinal properties included test-retest reliability (Intraclass Correlation Coefficient, ICC), correlation of change scores (Δ), and the accuracy of the PSQI-2 in detecting clinically meaningful change (ΔPSQI > 3) using the area under the curve (AUC).

**Results:**

The PSQI-2 showed a strong cross-sectional association with the full PSQI (β = 2.08, p < 0.001), explaining 72% of its variance. For identifying poor sleep quality, the PSQI-2 demonstrated excellent accuracy (AUC = 0.89) with an optimal cutoff of ≥ 2 (sensitivity = 77.5%, specificity = 84.5%). Longitudinally, both instruments showed moderate test-retest reliability (PSQI-2 ICC = 0.579; PSQI ICC = 0.639). The correlation between their change scores was strong (r = 0.682, p < 0.001), and the PSQI-2 showed reasonable accuracy (AUC = 0.716) in detecting clinically meaningful change (PSQI > 3).

**Conclusion:**

The PSQI-2 is a valid and reliable tool for cross-sectional screening of poor sleep quality in older men at the cutoff of ≥ 2. It is also responsive to directional change over time and can identify individuals with clinically significant changes in sleep.

## Background

Large-scale epidemiological studies are fundamental for investigating multiple health outcomes simultaneously, including cardiovascular diseases, neurodegenerative disorders, cancer incidence, musculoskeletal conditions, and mental health outcomes [1]. These studies face the significant challenge of collecting comprehensive data across these diverse domains while minimizing participant burden and maintaining high retention rates across multiple follow-ups. The necessity to cover numerous health themes often results in extensive questionnaires, which can lead to respondent fatigue, increased missing data, and reduced overall data quality [2]. Consequently, researchers are often forced to make difficult choices about which domains to include, and important health aspects, such as sleep quality, are frequently excluded or assessed with insufficient depth due to time and space constraints [3, 4]. In this context, the use of short, objective instruments becomes essential to ensure data quality and feasibility without compromising scientific rigor.

Sleep quality is a critical determinant of overall health and well-being, particularly in older adults [5]. Poor sleep is pervasive in this population and is intricately linked to a heightened risk of cognitive decline, cardiovascular disease, impaired physical function, and reduced quality of life [6, 7]. The Pittsburgh Sleep Quality Index (PSQI) is a well-validated instrument for assessing sleep quality, widely used [8], but its length (19 items) makes it impractical for certain large epidemiological investigations where multiple constructs need to be measured. This limitation is particularly relevant in studies involving older adults, where comprehensive assessment batteries can lead to respondent fatigue and increased missing data [2]. To address this challenge, abbreviated versions like the two-item PSQI (PSQI-2) have been developed, offering a pragmatic solution for sleep quality screening in resource-constrained scenarios [9].

The PSQI-2, derived from the components of sleep quality and sleep duration, has emerged as a promising short alternative [9]. Its brevity offers a compelling advantage for rapid screening. An initial cross-sectional study has demonstrated strong correlations between the PSQI-2 and the full PSQI, suggesting good concurrent validity [9]. However, despite its practical advantages, the psychometric properties of the scales require rigorous validation in different populations and contexts, particularly in large epidemiological studies with longitudinal designs. The MrOS Study provides an ideal platform for this validation, given its comprehensive sleep assessment protocol and well-characterized cohort of older men [10, 11].

Therefore, the main objective of this analysis was to conduct a comprehensive evaluation of the psychometric properties of the PSQI-2 in comparison with the full PSQI. Specifically, we aimed to: (1) confirm its cross-sectional concurrent validity and diagnostic accuracy for identifying poor sleep quality; (2) assess its test-retest reliability; and (3) evaluate its longitudinal responsiveness and ability to detect clinically meaningful changes in sleep quality over time.

## Methods

### Study population and design

This study used data from the Men’s Osteoporotic Fracture Sleep Study (MrOS), available from the Sleep Data platform. [11]. The MrOS Sleep Study was conducted between December 2003 and March 2005, during which 3,135 participants from the original cohort underwent a comprehensive sleep assessment constituting the study baseline (Visit 1). Exclusion criteria included regular use of positive airway pressure devices or supplemental oxygen during sleep. A follow-up examination (Visit 2) was conducted approximately 4.7 years (median) later, when sleep questionnaires were readministered. From the initial sleep study participants, 2,911 men with complete sleep questionnaire (PSQI) information formed our analytical sample. [10, 12, 13].

### Variables

#### Sleep Quality

The primary variables of interest were sleep quality measures. The PSQI was used to assess overall sleep quality. The global PSQI score, ranging from 0 to 21, was calculated from seven components: subjective sleep quality, sleep latency, sleep duration, habitual sleep efficiency, sleep disturbances, use of sleep medication, and daytime dysfunction, with higher scores indicating worse sleep quality [14]. The abbreviated PSQI-2 score was derived from the subjective sleep quality item (“During the past month, how would you rate your sleep quality overall?”) and the sleep duration component, based on the average hours of sleep per night. The scores from these two items (each ranging from 0–3) were summed to create a total score ranging from 0 to 6, where higher scores denote poorer sleep quality [9]. In a validation study, this instrument has demonstrated high internal consistency, with a Cronbach’s alpha of 0.94 (95%CI: 0.93–0.95) and McDonald’s ômega of 0.85 (95%CI: 0.84–0.86) [9].

#### Coviariates

A comprehensive array of covariates was employed to characterize the cohort and for potential adjustment in analyses. Sociodemographic factors included age, categorized into three groups (67–74, 75–84, and 85–90 years), self-reported race and ethnicity, and the level of educational attainment. Anthropometric measures consisted of body mass index, classified into standard categories of underweight, normal, overweight, and obese, and waist circumference, categorized based on established risk thresholds. Hypertension was defined based on measured blood pressure values and classified as present if systolic blood pressure ≥ 140 mmHg or diastolic blood pressure ≥ 90 mmHg [15]. Additional health status indicators included self-reported, physician-diagnosed morbidities: asthma, congestive heart failure, chronic obstructive pulmonary disease, diabetes, myocardial infarction, osteoarthritis, osteoporosis, and stroke.

Sleep-specific characteristics encompassed: (1) self-reported sleep disorders (insomnia, narcolepsy, periodic leg movements, restless legs, sleep apnea, or other sleep disorders); (2) objective polysomnography-derived metrics including apnea-hypopnea index, sleep efficiency, and percentage of sleep time in N3 and REM stages; and (3) validated sleep questionnaire scores assessing daytime sleepiness (ESS) [16], functional outcomes (FOSQ) [17], and insomnia severity (ISI) [18].

### Statistical analysis

The analytical strategy was comprehensive and multi-staged, incorporating both cross-sectional and longitudinal approaches to evaluate the psychometric properties of the PSQI-2. Data preparation involved cleaning and consistency checks, with the PSQI-2 score (range 0–6) as the index test and the full PSQI score (range 0–21) as the reference standard.

The primary cross-sectional analysis pooled data from both visits to maximize statistical power while accounting for within-subject correlation using mixed-effects models. Concurrent validity was assessed through repeated-measures correlation and mixed linear regression of the full PSQI on the PSQI-2. Agreement between the original PSQI and PSQI-2 instruments was then assessed using Bland-Altman analysis for repeated measures. To ensure optimal comparability between instruments, PSQI-2 scores were rescaled to match the PSQI’s 0–21 scale using linear regression coefficients derived from the relationship between the original measures. Diagnostic accuracy was assessed for established PSQI cutoffs (> 5, > 7, >10) using mixed-effects logistic regression. The area under the receiver operating characteristic curve (AUC) was calculated with cluster bootstrapping to derive confidence intervals. The optimal cutoff for the PSQI-2 was determined by maximizing Youden’s J index (J = sensitivity + specificity − 1), and sensitivity, specificity, positive and negative predictive values, and likelihood ratios were estimated at this threshold. Calibration was assessed by grouping predicted probabilities into deciles and plotting the mean predicted probability against the observed proportion of individuals with PSQI > 5, >7, and > 10 for each decile, with a nonparametric loess curve added to visualize the agreement relative to the 45-degree line of perfect calibration.

Secondary analyses were conducted separately for each visit to assess the temporal consistency of the psychometric properties. The longitudinal analysis focused on reliability and responsiveness. Test-retest reliability was quantified using the intraclass correlation coefficient (ICC) for absolute agreement from mixed models. Responsiveness to change was evaluated by calculating the correlation between change scores (Visit 2 – Visit 1) for the PSQI and PSQI-2 using Pearson coefficients. The standardized response mean (SRM) was calculated for each instrument. Furthermore, the ability of the absolute change in the PSQI-2 to detect a clinically meaningful change in the full PSQI, defined as an absolute change > 3 points, according to the literature [19, 20], was analyzed using ROC curves.

We also performed a binary logistic regression model to investigate which sociodemographic and health factors were associated with poor sleep quality according to the PSQI and PSQI-2 classifications. All analyses were performed using Stata version 18, employing robust standard errors where appropriate, and were two-sided with significance set at p < 0.05.

## Results

**Figure 1:**
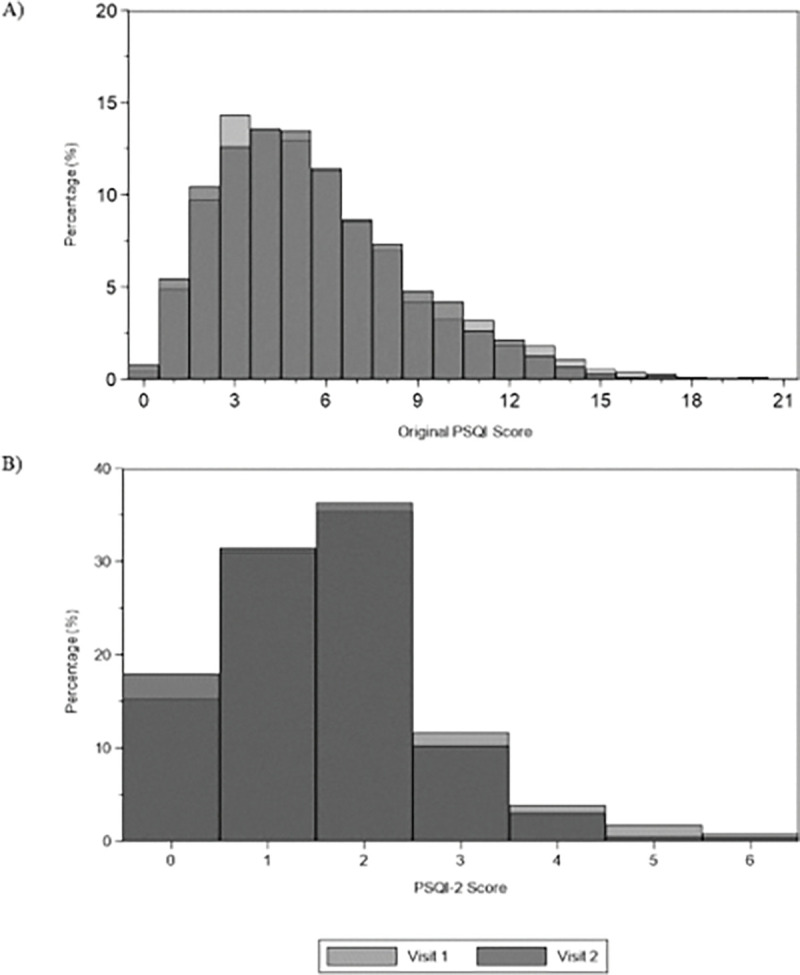
Distribution of PSQI and PSQI-2 scores in the MrOS Sleep Study. Histograms display the frequency distribution of the full PSQI total score (0–21) and the abbreviated PSQI-2 score (0–6) across all participants and both visits.

**Figure 2:**
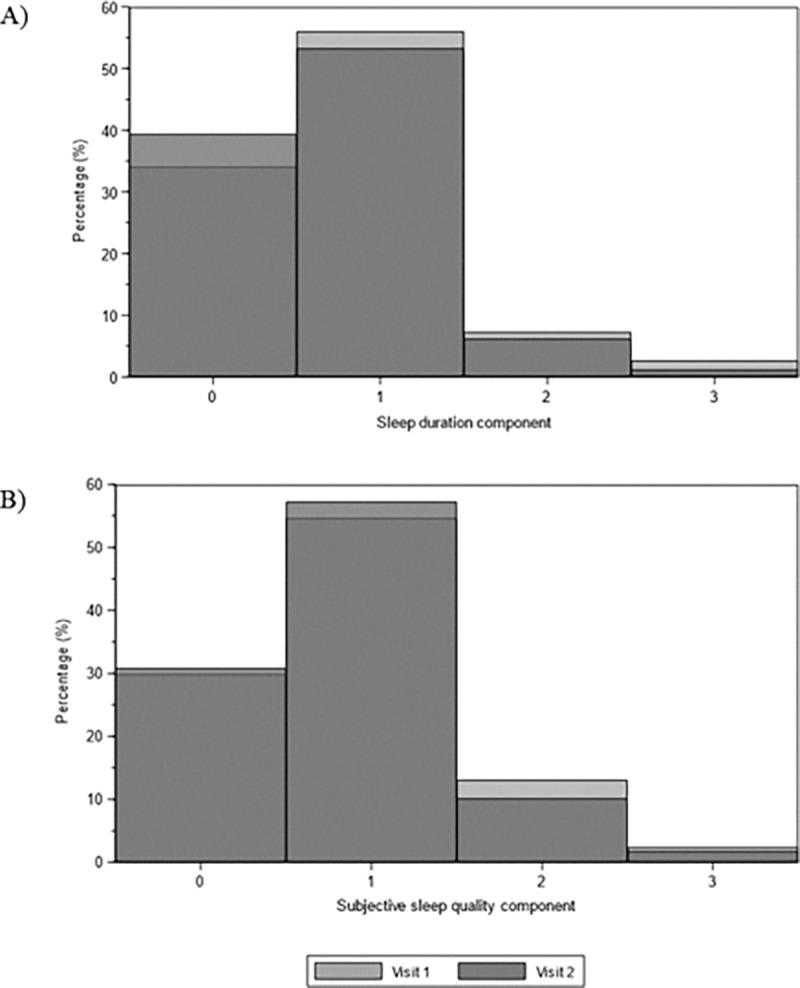
Distribution of PSQI-2 component scores by study visit in the MrOS Sleep Study. Histograms display the frequency distribution of (A) subjective sleep quality (score 0–3: very good to very poor), and (B) sleep duration component (score 0–3: ≥7 hours to <5 hours) across all participants and both visits.

The analysis included 2,911 men aged 67 to 90 years with complete data at both visits. As shown in Table 1, the cohort was predominantly White (90.7%) and well-distributed across educational, anthropometric, and clinical characteristics. Sleep quality scores remained stable between visits: the full PSQI averaged 5.63 (95% CI: 5.51–5.75) at Visit 1 and 5.48 (5.29–5.67) at Visit 2, while the PSQI-2 averaged 1.66 (1.62–1.71) and 1.52 (1.45–1.58), respectively. The prevalence of poor sleep (PSQI > 5) was consistent at 44.0% (Visit 1) and 43.6% (Visit 2), and for the PSQI-2 (PSQI-2 ≥ 2) was 53.6% (Visit 1) and 50.5% (Visit 2). Higher cutoffs (PSQI > 7 and > 10) also showed minimal change over time (Fig. 1). Furthermore, the distribution of PSQI-2 components remained stable across visits. For sleep quality (Fig. 2A), most participants reported “good” sleep (score 1: 54.7% V1, 57.4% V2). For sleep duration (Fig. 2B), most reported 6-<7 hours (score 1: 56.0% V1, 53.3% V2), with a 5.3% increase in optimal duration (≥ 7 hours) at V2. Confidence intervals overlapped for all categories, indicating no statistically significant changes between visits.

**Figure 3:**
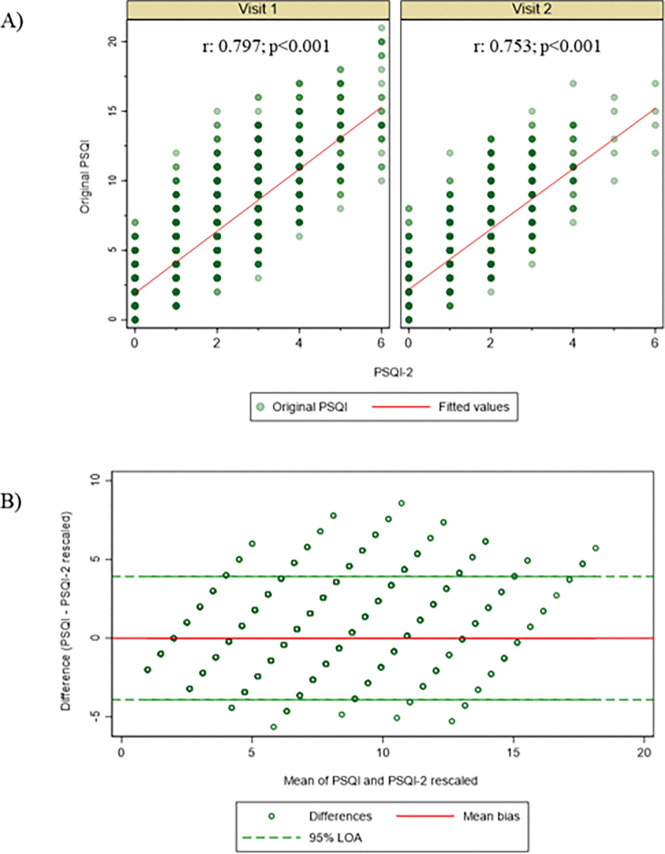
Correlation between PSQI-2 and PSQI total scores across both visits of the MrOS Sleep Study and Bland–Altman plots comparing PSQI-2 with PSQI total scores Panel A shows a scatterplot of PSQI-2 (x-axis) versus PSQI total (y-axis), with fitted regression line and confidence bands, demonstrating strong concurrent validity. Each dot represents one observation from one visit. Panel B displays Bland–Altman plots, with the mean of the two measures (x-axis) plotted against their difference (y-axis). Solid lines represent mean bias and dashed lines the 95% limits of agreement. The PSQI-2 score was rescaled to the original PSQI metric (0–21) using the linear regression equation derived from the data: PSQI-2 Rescaled = 1.99 + 2.21 × PSQI-2.

The cross-sectional pooled analysis demonstrated a strong concurrent validity between the PSQI-2 and the full PSQI, with the full PSQI, with Pearson correlations exceeding 0.70 across both visits (Fig. 2). The mixed-effects model revealed a highly significant association (β = 2.167, p < .001) (Fig. 3A). The repeated-measures correlation coefficient was 0.786. The Bland-Altman analysis revealed excellent agreement. Linear regression-based rescaling eliminated the systematic bias between the original PSQI and the PSQI-2, resulting in a mean difference of 0.00 points. The agreement limits of −3.91 to 3.91 correspond to approximately 18% of the total scale (0–21) (Fig. 3B).

**Figure 4:**
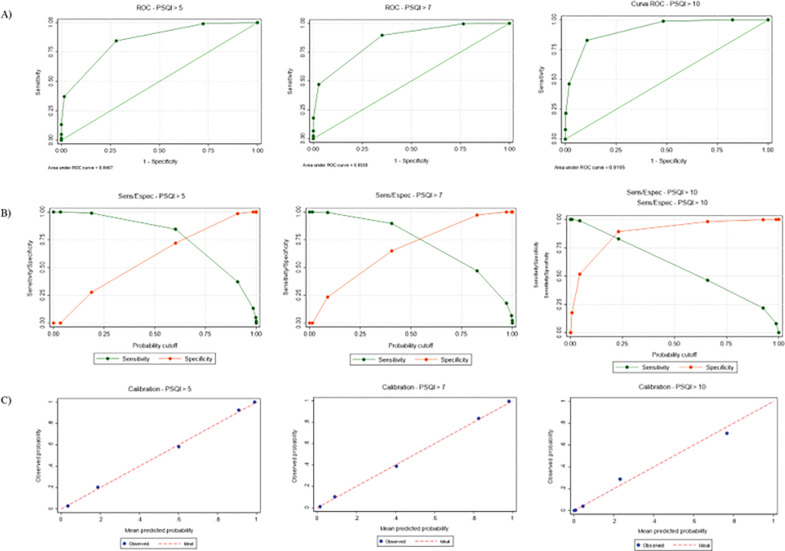
Diagnostic accuracy of the PSQI-2 for detecting poor sleep quality defined by PSQI thresholds Panel A presents ROC curves of the PSQI-2 against three PSQI cut-offs (>5, >7 and >10). Area under the curve (AUC) with 95% confidence intervals is provided. Panel B shows sensitivity and specificity curves across different probability thresholds, illustrating the trade-off between these indicators. Panel C displays calibration plots comparing observed versus predicted probabilities for each cut-off, with the diagonal representing perfect calibration. The figure demonstrates that the PSQI-2 achieves good to excellent diagnostic accuracy across cut-offs, with AUC values ranging from 0.85 to 0.92, and shows adequate calibration.

In terms of diagnostic accuracy, ROC analyses showed that the PSQI-2 achieved good performance across conventional cut-points, with AUC values consistently above 0.80 (Fig. 4A). At the traditional threshold of PSQI > 5, the optimal PSQI-2 cut-point was ≥ 2, yielding balanced sensitivity (84.5%) and specificity (72.0%). Similar results were observed for the other cutoffs (> 7 and > 10), with detailed metrics provided in Fig. 4B. The calibration plot for the prediction of PSQI > 5, > 7, and > 10 indicates very good agreement between the predicted probabilities from the model and the observed outcomes. The non-parametric loess curve (representing the observed probabilities) closely follows the ideal 45-degree line of perfect calibration across almost the entire range of predicted probabilities (Fig. 4C).

**Figure 5:**
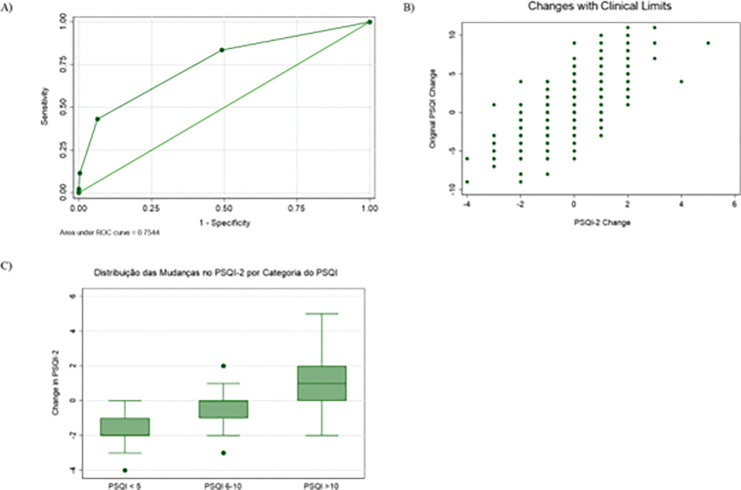
Responsiveness and longitudinal validity of the PSQI-2 compared with the PSQI. Panel A shows ROC curves for the ability of PSQI-2 change scores to detect clinically meaningful improvement in sleep quality, defined as a >3-point reduction in PSQI total score (AUC=0.75). Panel B illustrates scatterplots of change in PSQI-2 versus change in PSQI, with vertical and horizontal reference lines marking clinical thresholds (±1 for PSQI-2 and ±3 for PSQI), classifying individuals as improved, stable, or worsened. Panel C displays boxplots of PSQI-2 change according to categories of PSQI change (<5, 6–10, >10), highlighting expected directional patterns.

The longitudinal analysis revealed moderate test-retest reliability for both instruments. The ICC for the full PSQI was 0.639, while for the PSQI-2 it was 0.579, indicating acceptable but not excellent stability over time. The responsiveness analysis showed a strong correlation between the change scores of the two instruments (Pearson’s r = 0.682, p < .001; Spearman’s rho = 0.652, p < .001), confirming that both scales capture similar directions of change in sleep quality. However, the agreement in categorical classification of change (improved, stable, worsened) was only fair (Kappa = 0.351), despite a 76.2% exact agreement. The SRM was small for both the original PSQI (0.07) and the PSQI-2 (−0.13). The ROC analysis for detecting a clinically significant change (> 3 points on the full PSQI) yielded an AUC of 0.716 for the absolute change in PSQI-2, demonstrating reasonable accuracy (Fig. 5).

Figure 5, furthermore, illustrates the relationship between individual changes on both instruments, incorporating clinical thresholds. This scatterplot (Fig. 5B), with reference lines at ± 3 points for the PSQI (vertical) and ± 1 point for the PSQI-2 (horizontal), shows a general diagonal trend indicating concordance in the direction of change. This interpretation is supported by Fig. 3C, a boxplot showing the distribution of PSQI-2 change scores stratified by the categorical change in the full PSQI (using the > 3 point threshold). The medians align with expectations, negative for the “improved” group, near zero for the “stable” group, and positive for the “worsened” group, but the variability, especially within the “improved” and “worsened” categories, underscores the differences in sensitivity between the two measures.

Associations between poor sleep quality and sociodemographic/clinical factors are shown in Table 2. Both instruments identified consistent risk factors, including cardiovascular conditions (congestive heart failure: PSQI OR = 1.68, PSQI-2 OR = 1.60), respiratory disease (COPD: PSQI OR = 1.68, PSQI-2 OR = 1.50), and osteoarthritis (PSQI OR = 1.60, PSQI-2 OR = 1.29). Graduate education was protective on both scales (PSQI OR = 0.69, PSQI-2 OR = 0.72). The overlapping confidence intervals for nearly all estimates indicate no significant differences between instruments in detecting these associations, except FOSQ scores, where the full PSQI showed a stronger association; however, both in the same direction (OR: 0.66; 95%CI: 0.62–0.70 and OR: 0.77; 95%CI: 0.73–0.82, for PSQI and PSQI-2, respectively).

## Discussion

This study provides a comprehensive longitudinal evaluation of the abbreviated two-item Pittsburgh Sleep Quality Index (PSQI-2) against the full PSQI in a large cohort of community-dwelling older men. Our findings confirm strong cross-sectional validity and excellent diagnostic accuracy of the PSQI-2, supporting its use as an efficient screening tool. However, the analysis also reveals nuances regarding its longitudinal performance, highlighting both its capabilities and limitations for tracking changes in sleep quality over time.

The robust cross-sectional association between the PSQI-2 and the full PSQI demonstrates that the two items, subjective sleep quality and sleep duration, capture the majority of the variance in the global PSQI score. This finding is consistent with previous validation studies in other populations. Famodu et al. (2018) [21], for example, developed a reduced 13-item version, maintaining a high correlation with the original PSQI (rho = 0.94), while Sancho-Domingo et al. (2021)[22] Validated a 6-item version with a correlation of 0.93 and high accuracy (AUC = 0.87) for identifying sleep disorders. Furthermore, the study of the proposed and validation of PSQI-2, with adults in a population-based household survey with stratified sampling in Brazil, had excellent internal consistency and known-group validity, with a sensitivity of 77.9% and specificity of 73.8% [9].

The selection of the two components of the PSQI-2, subjective sleep quality and sleep duration, is supported by guidelines from organizations such as the National Sleep Foundation and the American Academy of Sleep Medicine, which recognize these domains as central to the assessment of sleep health. [23]. Previous psychometric studies have shown that these items represent the core constructs of the complete PSQI [9]. Previous factor analyses [9]Have identified that these components capture essential dimensions of sleep, perceptual, and behavioral, with robust factor loadings and adequate internal consistency.

Our results corroborate the validity of this abbreviated approach. The strong correlation between the PSQI-2 and the full instrument, together with its excellent diagnostic accuracy, demonstrates that these two items preserve the screening capacity of the original instrument. Moderate longitudinal stability and the ability to detect clinically significant changes reinforce its usefulness in longitudinal contexts, suggesting that while there is a degree of natural variability in self-reported sleep over one year, the PSQI-2 is reasonably stable. Furthermore, the PSQI-2 demonstrated a reasonable ability to detect those who experienced a clinically meaningful change, defined as a > 3 point shift on the full PSQI [19, 20]. This suggests that while the absolute change measured by the PSQI-2 may not directly mirror that of the full version, it retains significant utility for identifying individuals whose sleep has substantively improved or declined, a key requirement for patient management and research outcomes.

Although brief instruments offer advantages in terms of practicality and reduced burden on respondents [24]It is essential to consider their limitations. Abbreviated versions may require context- and population-specific cut-off points, and do not capture multidimensionality, such as sleep disturbances, medication use, and daytime dysfunction [14], which additionally increase the total score but are not fully captured in the two-item version. However, our results demonstrated that the PSQI-2 maintained consistent performance, supporting its use as a balanced tool between brevity and validity for large-scale studies and clinical screening.

Several limitations must be considered. The study cohort consisted exclusively of older men, predominantly white, which may limit the generalizability of our findings to women or more ethnically diverse populations. Furthermore, the definition of “clinically meaningful change” was anchored to the full PSQI rather than an external clinical anchor. Future studies would benefit from using such external criteria to further validate the responsiveness of the PSQI-2. Among the strengths, note the longitudinal design with two assessments, which allowed us to analyze not only cross-sectional validity but also the reliability and responsiveness of the instrument over time. The large sample size and comprehensive characterization of the cohort, with detailed data on general health, comorbidities, and objective sleep parameters, allowed for robust analyses. The use of statistical methods appropriate for longitudinal data, such as mixed-effects models and repeated measures correlation analysis, reinforces the validity of the conclusions. Finally, the simultaneous assessment of multiple psychometric properties, including concurrent validity, reliability, diagnostic accuracy, and responsiveness, provides a comprehensive view of the PSQI-2’s performance in a real-life context.

## Conclusion

This study demonstrates that the PSQI-2 is a robust and practical instrument for assessing sleep quality in older men. Our findings confirm its strong concurrent validity with the full PSQI, excellent diagnostic accuracy for identifying poor sleep quality (PSQI > 5) at the optimal cutoff of ≥ 2, and moderate test-retest reliability. The full PSQI remains the standard index for a comprehensive, multidimensional assessment. The PSQI-2’s strength lies in its ability to efficiently track directional changes and classify poor sleepers, making it particularly valuable for large-scale epidemiological studies, longitudinal monitoring, and initial clinical screening.

Future research should evaluate these findings in more diverse populations, including women and other ethnic groups. Nevertheless, based on our results, the PSQI-2 can be confidently recommended as a valid, reliable, and responsive brief measure of sleep quality in aging populations, in both clinical and research settings where the full PSQI may be impractical.

## Figures and Tables

**Table 1:** Sociodemographic, anthropometric, and clinical characteristics of the MrOS Sleep participants according to poor sleep quality defined by the PSQI and the PSQI-2

	Total				PSQI				PSQI 2			
	n	%	95%CI		n	%	95%CI		n	%	95%CI	
**Age**												
67–74 years old	1247	42.8	41.0	44.6	538	43.2	40.4	45.9	673	54.0	51.3	56.8
75–84 years old	1404	48.2	46.4	50.1	620	44.2	41.6	46.8	756	53.9	51.2	56.5
85–90 years old	260	8.9	7.9	10.0	123	47.3	41.2	53.4	130	50.2	44.1	56.3
**Race/Ethnicity**												
African American	99	3.4	2.7	4.1	49	49.5	39.7	59.3	61	61.6	52.0	71.2
American Indian or Alaska Native	32	1.1	0.7	1.5	16	50.0	32.7	67.3	14	43.8	26.6	60.9
Asian	84	2.9	2.3	3.5	42	50.0	39.3	60.7	53	63.1	52.8	73.4
Native Hawaiian or Other Pacific Islander	55	1.9	1.4	2.4	29	52.7	39.5	65.9	32	58.2	45.2	71.2
White	2641	90.7	89.7	91.8	1145	43.4	41.5	45.3	1399	53.0	51.1	54.9
**Initial education level**												
High school or less	624	21.4	20.0	22.9	311	49.8	45.9	53.8	366	58.7	54.8	62.5
Some college/college graduate	1178	40.5	38.7	42.3	529	44.9	42.1	47.8	640	54.4	51.6	57.3
Graduate degree	1109	38.1	36.3	39.9	441	39.8	36.9	42.7	553	49.9	46.9	52.8
**Anthropometry**												
**Body mass index**												
Normal	1463	50.3	48.5	52.1	628	42.9	40.4	45.5	771	52.7	50.1	55.3
Underweight	234	8.0	7.1	9.0	99	42.5	36.1	48.8	122	52.6	46.2	59.0
Overweight	704	24.2	22.6	25.8	307	43.6	39.9	47.3	378	53.7	50.0	57.4
Obese	508	17.5	16.1	18.8	245	48.2	43.9	52.6	286	56.3	52.0	60.6
**Waist circumference**												
Normal	1827	62.9	61.2	64.7	782	42.8	40.6	45.1	956	52.4	50.1	54.7
Increased	726	25.0	23.4	26.6	312	43.0	39.4	46.6	392	54.0	50.4	57.6
Very increased	351	12.1	10.9	13.3	183	52.1	46.9	57.4	206	58.7	53.5	63.8
**Hypertension**												
Normotensive	2333	80.1	78.7	81.6	1019	43.7	41.7	45.7	1258	54.0	51.9	56.0
Hypertensive	578	19.9	18.4	21.3	262	45.3	41.3	49.4	301	52.1	48.0	56.2
**Morbidities**												
Asthma	224	7.7	6.7	8.7	106	47.3	40.8	53.9	127	56.7	50.2	63.2
Congestive heart failure or enlarged heart	174	6.0	5.1	6.8	93	53.5	46.0	60.9	110	63.2	56.1	70.4
COPD, chronic obstructive lung disease, or emphysema	151	5.2	4.4	6.0	85	56.3	48.4	64.2	95	62.9	55.2	70.6
Diabetes	387	13.3	12.1	14.5	184	47.6	42.6	52.5	221	57.1	52.2	62.0
Heart attack, coronary or myocardial infarction	508	17.5	16.1	18.8	270	53.2	48.8	57.5	303	59.7	55.4	63.9
Osteoarthritis or degenerative arthritis	701	24.1	22.5	25.7	373	53.2	49.5	56.9	414	59.1	55.4	62.7
Osteoporosis	212	7.3	6.3	8.2	108	50.9	44.2	57.7	125	59.0	52.3	65.6
Stroke, blood clot in the brain or bleeding in the brain	111	3.8	3.1	4.5	61	55.0	45.7	64.2	64	57.7	48.5	66.9
**Sleep disorders**												
None	2631	90.4	89.3	91.5	1107	42.1	40.2	44.0	1364	51.9	50.0	53.8
At least one	280	9.6	8.6	10.7	174	62.1	56.5	67.8	195	69.6	64.3	75.0
**Sleep architecture**												
Apnea-hypopnea index	-	18.3	17.7	18.8	-	18.7	17.8	19.6	-	18.65	17.9	19.5
Sleep efficiency (%)	-	76.0	75.6	76.5	-	74.3	73.6	75.0	-	75.03	74.4	75.7
Stage N3 of sleep (%)	-	11.3	10.9	11.6	-	11.1	10.6	11.6	-	11.34	10.9	11.8
Stage REM of sleep (%)		19.3	19.0	19.5	-	19.0	18.6	19.3	-	19.02	18.7	19.4
**Sleep questionnaires**												
ESS score	-	6.2	6.0	6.3	-	6.6	6.4	6.8	-	6.49	6.3	6.7
FOSQ score		18.7	18.7	18.8	-	18.2	18.1	18.3	-	18.45	18.4	18.5
ISI score	-	4.9	4.7	5.2	-	7.4	7.0	7.9	-	6.84	6.4	7.2

This table presents the distribution of poor sleep quality across subgroups defined by age, race/ethnicity, education, body mass index, waist circumference, hypertension, chronic conditions, and sleep disorders. Poor sleep was defined as PSQI total score > 5 and PSQI-2 score ≥ 2. Prevalence estimates (%) are shown with 95% confidence intervals.

**Table 2:** Associations of sociodemographic, anthropometric, and clinical characteristics with poor sleep quality according to the PSQI and the PSQI-2

	PSQI				PSQI 2				Difference PSQI vs PSQI2
	OR	95%CI		p-value	OR	95%CI	p-value		
**Age**									
67–74 years old	1.00	-	-	-	1.00	-	-	-	-
75–84 years old	1.03	0.90	1.18	0.684	0.96	0.84	1.11	0.612	No sig
85–90 years old	1.09	0.88	1.34	0.427	0.78	0.63	0.97	0.022	No sig
**Race/Ethnicity**									
African American	1.00	-	-	-	1.00	-	-	-	-
American Indian or Alaska Native	1.41	1.00	1.98	0.048	1.49	1.05	2.11	0.024	No sig
Asian	1.03	0.72	1.46	0.880	1.41	0.99	2.02	0.056	No sig
Native Hawaiian or Other Pacific Islander	1.71	1.10	2.66	0.016	1.39	0.89	2.17	0.144	No sig
White	1.11	0.63	1.96	0.722	0.72	0.40	1.27	0.252	No sig
**Initial education level**									
High school or less	1.00	-	-	-	1.00	-	-	-	-
Some college/college graduate	0.85	0.72	1.01	0.065	0.84	0.71	1.00	0.052	No sig
Graduate degree	0.69	0.58	0.82	0.000	0.72	0.61	0.86	0.000	No sig
**Anthropometry**									
**Body mass index**									
Normal	1.00	-	-	-	1.00	-	-	-	-
Underweight	0.92	0.73	1.16	0.470	0.89	0.71	1.12	0.330	No sig
Overweight	1.11	0.95	1.30	0.187	1.04	0.89	1.21	0.656	No sig
Obese	1.38	1.15	1.64	0.000	1.14	0.96	1.36	0.143	No sig
**Waist circumference**									
Normal	1.00	-	-	-	1.00	-	-	-	-
Increased	1.06	0.91	1.23	0.451	1.03	0.89	1.19	0.719	No sig
Very increased	1.47	1.21	1.79	0.000	1.25	1.03	1.53	0.025	No sig
**Hypertension**									
Normotensive	1.00	-	-	-	1.00	-	-	-	-
Hypertensive	1.02	0.90	1.16	0.744	0.89	0.78	1.01	0.076	No sig
**Morbidities**									
Asthma	1.24	0.98	1.57	0.080	1.18	0.93	1.50	0.170	No sig
Congestive heart failure or enlarged heart	1.68	1.29	2.18	0.000	1.60	1.22	2.09	0.001	No sig
COPD, chronic obstructive lung disease, or emphysema	1.68	1.21	2.34	0.002	1.50	1.07	2.10	0.019	No sig
Diabetes	1.21	1.01	1.45	0.041	1.10	0.92	1.32	0.311	No sig
Heart attack, coronary or myocardial infarction	1.55	1.31	1.83	0.000	1.30	1.10	1.54	0.002	No sig
Osteoarthritis or degenerative arthritis	1.60	1.38	1.86	0.000	1.29	1.11	1.49	0.001	No sig
Osteoporosis	1.31	1.04	1.66	0.022	1.20	0.95	1.52	0.125	No sig
Stroke, blood clot in the brain or bleeding in the brain	1.40	1.02	1.92	0.040	1.02	0.74	1.40	0.925	No sig
**Sleep disorders**									
None	1.00	-	-	-	1.00	-	-	-	-
At least one	2.02	1.66	2.47	0.000	1.82	1.49	2.23	0.000	No sig
**Sleep architecture**									
Apnea-hypopnea index	1.00	1.00	1.01	0.106	1.00	1.00	1.01	0.086	No sig
Sleep efficiency (%)	0.98	0.98	0.99	0.000	0.99	0.98	0.99	0.000	No sig
Stage N3 of sleep (%)	1.00	0.99	1.00	0.289	1.00	1.00	1.01	0.256	No sig
Stage REM of sleep (%)	0.99	0.98	0.99	0.002	0.99	0.98	1.00	0.069	No sig
**Sleep questionnaires**									
ESS score	1.05	1.03	1.07	0.000	1.05	1.03	1.07	0.000	No sig
FOSQ score	0.66	0.62	0.70	0.000	0.77	0.73	0.82	0.000	Sig
ISI score*	1.37	1.31	1.44	0.000	1.31	1.26	1.37	0.000	No sig

Odds ratios (OR) and 95% confidence intervals from logistic regression models are reported, comparing poor versus good sleepers according to the PSQI (> 5) and PSQI-2 (≥ 2).

## Data Availability

The datasets analyzed for this study were provided by the National Sleep Research Resource (Sleep Data, https://sleepdata.org). The MrOS data are publicly available for researchers upon request. Access to the data requires approval of a data use agreement and compliance with the terms and conditions set by the MrOS Study and the Sleep Data platform.
